# Sociodemographic Disparities in Pediatric Dermatological Conditions: Insights From the 2019 Global Burden of Disease Project

**DOI:** 10.1002/pdi3.70043

**Published:** 2026-03-27

**Authors:** Elyse Mackenzie, Nicole Dib, Sydney Wolfe, Rose Rasty, Bianca Sanabria, Shilpa Pai

**Affiliations:** ^1^ Rutgers Robert Wood Johnson Medical School Piscataway New Jersey USA; ^2^ Department of Pediatrics Rutgers Robert Wood Johnson Medical School Piscataway New Jersey USA

## 1 Introduction

Skin and subcutaneous diseases are among the leading contributors to nonfatal health outcomes globally, accounting for over 42 million disability‐adjusted life years (DALYs) worldwide [1]. This burden is particularly concerning in pediatric populations, where dermatologic conditions can affect long‐term physical and psychosocial health outcomes [2]. By understanding the interplay of age, environmental exposure, and sociodemographic factors, clinicians and policymakers can develop interventions tailored to the needs of specific pediatric populations.

Existing research highlights the influence of developmental stages, such as the atopic march and pubertal changes, on the prevalence of skin diseases [3]. However, the role of sociodemographic factors—including access to healthcare, sanitation, and education—remains underexplored in determining pediatric dermatologic outcomes [4]. Using data from the 2019 Global Burden of Disease (GBD) study [1], this study aims to describe patterns of pediatric skin disease prevalence across sociodemographic contexts, emphasizing the need for targeted public health strategies to address observed disparities.

## 2 Methods

The 2019 GBD study offers epidemiologic insights regarding the global impact and distribution of skin disease [1]. Rather than drawing from a discrete sample size, GBD 2019 DALY rates are derived from 281,586 input sources across 204 countries and territories, integrated through standardized modeling frameworks [1]. Data was extracted from the GBD interactive data visualization tool for all available conditions related to skin and subcutaneous diseases, including malignant skin melanoma, nonmelanoma skin cancer, dermatitis, psoriasis, bacterial skin diseases, scabies, fungal skin diseases, viral skin diseases, acne vulgaris, alopecia areata, pruritus, urticaria, decubitus ulcer, and “other.”

Disease prevalence was analyzed for age groups 0–14 years (children) and 15–19 years (adolescents) for male and female sexes. These age ranges, predetermined by the GBD, offer a general distinction between earlier and later pubertal stages. Prevalence data, reported as a percentage, was further extracted across three levels of the sociodemographic index (SDI): low, medium, and high. SDI, a composite measure of income, education, and fertility, reflects a region's overall health‐related resources. For each age group and SDI category, the five most prevalent conditions were recorded and presented in a combined table (Table 1).

**TABLE 1:** Skin disease prevalence by SDI level and age group.

Rank	Age group (years)	Low SDI (%)	Medium SDI (%)	High SDI (%)
1	0–14	Fungal skin diseases (15.78)	Fungal skin diseases (5.72)	Atopic Dermatitis (9.90)
	15–19	Acne vulgaris (15.11)	Acne vulgaris (15.72)	Acne vulgaris (19.05)
2	0–14	Viral skin disease (4.29)	Other (5.09)	Viral skin disease (7.45)
	15–19	Fungal skin disease (9.37)	Other (5.73)	Other (6.67)
3	0–14	Other (3.61)	Scabies (4.42)	Other (5.40)
	15–19	Other (4.29)	Fungal skin disease (5.60)	Atopic Dermatitis (4.75)
4	0–14	Atopic Dermatitis (3.13)	Viral skin disease (4.22)	Acne vulgaris (4.44)
	15–19	Atopic Dermatitis (3.00)	Scabies (4.50)	Viral skin disease (4.47)
5	0–14	Acne vulgaris (2.87)	Atopic Dermatitis (3.95)	Fungal skin disease (3.14)
	15–19	Scabies (2.56)	Atopic Dermatitis (3.43)	Fungal skin disease (3.36)

*Note:* Prevalence (%) of the five most common pediatric skin and subcutaneous diseases in 2019, stratified by sociodemographic index (SDI) level and age group (0–14 years, 15–19 years). “Rank” indicates order of prevalence within each category (Rank 1 = most prevalent; Rank 5 = fifth most prevalent). “Other” refers to aggregated skin and subcutaneous conditions not individually classified by the Global Burden of Disease (GBD) study.

Abbreviation: SDI, sociodemographic index.

## 3 Results

In low SDI regions, fungal skin diseases were most common in children (15.78%), whereas acne vulgaris predominated among adolescents (15.11%). In medium SDI regions, fungal skin diseases remained the most prevalent condition in children (5.72%), whereas acne vulgaris was most common in adolescents (15.72%). High SDI regions revealed a different trend, with dermatitis most common in children (9.90%), whereas acne vulgaris remained the most prevalent among adolescents (19.05%). Detailed prevalence rates are shown in Table 1.

## 4 Discussion

The GBD 2019 study reveals distinct patterns in pediatric dermatological conditions across sociodemographic contexts. Among children, fungal skin diseases were most prevalent in low SDI regions, which may be due to environmental factors such as poor sanitation and low hygiene education [4]. These findings suggest the need for interventions focused on improving sanitation infrastructure and public health campaigns promoting hygiene practices.

In adolescents, acne vulgaris emerged as the most prevalent condition in all SDI levels but was particularly prominent in high SDI regions. This trend may reflect dietary patterns characterized by high refined carbohydrates and fats, rising obesity rates, and elevated stress related to academic and social pressures [3]. Additionally, improved healthcare infrastructure and access to dermatological care in high SDI regions likely contributes to higher diagnostic and reporting rates [3]. These findings underscore the need for targeted programs addressing dietary education, stress management, and mental health resources in adolescent patients in these areas.

Interestingly, the consistent prevalence of atopic dermatitis across all SDI levels suggests that genetic predisposition and environmental allergens may play more significant roles in disease development than SDI. Notably, higher income has been associated with increased prevalence of atopic dermatitis, a pattern often attributed to the hygiene hypothesis. This theory suggests that reduced early‐life exposures to infections in higher‐income settings may hinder immune maturation and increase susceptibility to atopy, whereas more frequent infections in lower income or rural settings may decrease the risk [5].

Although these findings provide valuable insights, several limitations warrant consideration. The broad age groupings (0–14 years and 15–19 years) may obscure important differences within subgroups, such as infants, toddlers, and early adolescents. Additionally, the reliance on aggregated GBD data may overlook regional nuances and inconsistencies in data collection methods. Future research should aim to stratify data by narrower age groups and incorporate localized studies to better understand these trends.

## 5 Conclusion

This study highlights the complex relationship between sociodemographic factors and pediatric dermatological outcomes, emphasizing the need for tailored public health interventions. In low SDI regions, efforts should prioritize sanitation and hygiene education to address fungal infections in young children. In high SDI regions, addressing dietary habits and stress‐related factors in adolescents could mitigate the burden of acne vulgaris. By integrating sociodemographic considerations into healthcare strategies, stakeholders can better address disparities and improve dermatological outcomes for pediatric populations worldwide.

## Author Contributions

E.M. and N.D. contributed to study conception, design, and manuscript writing. S.W. and R.R. contributed to data extraction and assisted with manuscript preparation. B.S. contributed to study conception and design and provided critical manuscript editing. S.P. served as senior author, overseeing the project and supervising manuscript development.

## Funding

This study received no funding.

## Conflicts of Interest

The authors declare no conflicts of interest.

## Data Availability

This article utilized data that is publicly available from the Global Burden of Disease data, which is freely accessible through https://vizhub.healthdata.org/gbd-results/.
